# Truncated Power Laws Reveal a Link between Low-Level Behavioral Processes and Grouping Patterns in a Colonial Bird

**DOI:** 10.1371/journal.pone.0001992

**Published:** 2008-04-23

**Authors:** Roger Jovani, David Serrano, Esperanza Ursúa, José L. Tella

**Affiliations:** Department of Conservation Biology, Estación Biológica de Doñana, Consejo Superior de Investigaciones Científicas (CSIC), Pabellón del Perú, Sevilla, Spain; University of Utah, United States of America

## Abstract

**Background:**

Departures from power law group size frequency distributions have been proposed as a useful tool to link individual behavior with population patterns and dynamics, although examples are scarce for wild animal populations.

**Methodology/Principal Findings:**

We studied a population of Lesser kestrels (*Falco naumanni*) breeding in groups (colonies) from one to ca. 40 breeding pairs in 10,000 km^2^ in NE Spain. A 3.5 fold steady population increase occurred during the eight-year study period, accompanied by a geographical expansion from an initial subpopulation which in turn remained stable in numbers. This population instability was mainly driven by first-breeders, which are less competitive at breeding sites, being relegated to breed solitarily or in small colony sizes, and disperse farther than adults. Colony size frequency distributions shifted from an initial power law to a truncated power law mirroring population increase. Thus, we hypothesized that population instability was behind the truncation of the power law. Accordingly, we found a power law distribution through years in the initial subpopulation, and a match between the power law breakpoint (at ca. ten pairs) and those colony sizes from which the despotic behavior of colony owners started to impair the settlement of newcomers. Moreover, the instability hypothesis was further supported by snapshot data from another population of Lesser kestrels in SW Spain suffering a population decline.

**Conclusions/Significance:**

Appropriate analysis of the scaling properties of grouping patterns has unraveled the link between local agonistic processes and large-scale (population) grouping patterns in a wild bird population.

## Introduction

After decades of parallel research in animal behavior and population ecology, studying the link between them is key to advance in the understanding of many natural phenomena [1]–[5]. A particularly active area of research is the study of animal grouping patterns. Modeling approaches of presumed generic grouping dynamics have shown that simple and homogeneous local level interactions (e.g. groups tend to aggregate when they meet) could lead to large-scale heterogeneities in population grouping patterns similar to those found in nature [6]. Empirical studies conducted under laboratory conditions, where both individual behavior details and statistical grouping patterns and dynamics have been studied, further support this link between small-scale processes and population patterns [7]–[10]. However, despite the importance of this issue, empirical evidence is still scarce for wild animal populations, and particularly for birds. This is probably due to logistic problems derived from studying simultaneously fine-scale behavioral processes and large-scale population patterns and dynamics, but also because adequate analytical frameworks are still lacking.

Recently, Sjöberg *et al*. [11] have suggested that truncated power laws (two different power law regimes joined by a breakpoint) could be a promising tool to achieve this goal. Power laws describe relationships that hold at different scales [12] (see methods). In this way, ruptures of this scale invariance have been interpreted as potential fingerprints of relevant processes acting within the system [6], [11], such as the abundance of resources [13], the availability of suitable habitat [11], or the presence of mutualistic species [14]. In this study we examine bird colony sizes, which were suggested [15], and recently confirmed in a bird species [16], to follow power law frequency distributions. Here, we used power laws as a null model of colony size frequency distributions. Then, we explored potential truncations of this hypothetical distribution as a tool to hypothesize and test the effect of internal population processes on these population patterns [11]. We did so studying a bird population from which we had previous detailed information thanks to a log-term monitoring of the colony size and breeding success of all the colonies in the population, and the ringing and following of movements and other behaviors of thousands of individual birds [17]–[20].

**Figure 1:**
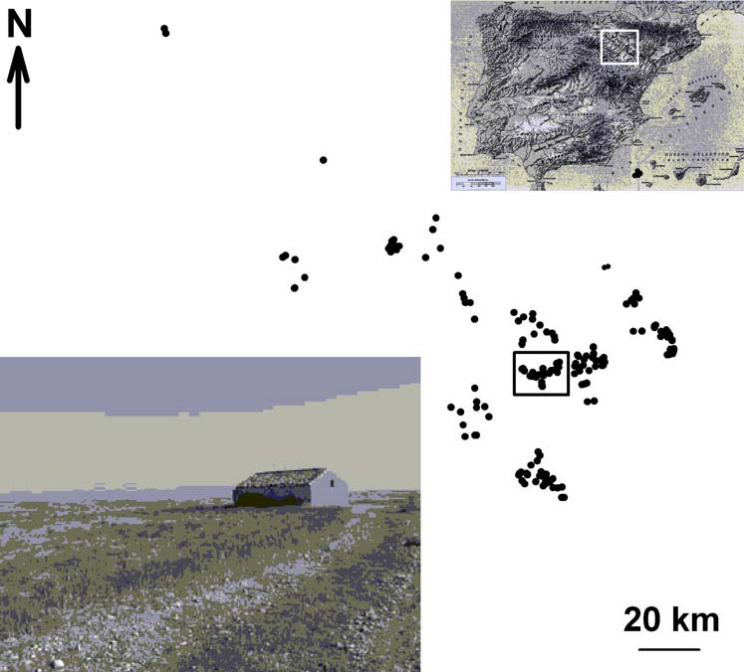
Location of Lesser kestrel colonies (farmhouses) in the study area in year 2000. The rectangle encompasses the initial subpopulation named Sastago. Inset pictures show the location of the study area (Ebro Valley, NE Spain), and an example of one of the farmhouses where these small falcons bred under roof tiles.

The study system is a population of a small-sized raptor (the Lesser kestrel, *Falco naumanni*) that extends 10,000 km^2^ along the Ebro Valley (NE Spain) (Figure 1). There, birds breed in groups (hereafter colonies) (see methods) from 1 to ca.40 nests (i.e. breeding pairs) occurring under tiled roofs of small and isolated farmhouses (Figure 1). Lesser kestrels do not exclude other individuals from their feeding areas around the colonies, but defend vigorously the nest-site and its surroundings from conspecifics. Hence, due to the small size of the roofs (*ca*. 50 m^2^), large colonies often become hostile crowded places [17], [18]. These large colonies are mostly composed of adult birds, which are very faithful to the colony between consecutive years and win most aggressive encounters against prospecting (mainly juvenile) birds [17]–[20]. In fact, most juvenile birds are often forced to leave the colony after a chain of successive attacks from nest owners. In this way, although prospectors are particularly attracted to large colonies, they finally settle in smaller colonies [18], [20]. In summary, juveniles possess poorer competitive skills [18] and move longer distances than adults [19], [20], so they tend to join small colonies and create new ones.

**Figure 2:**
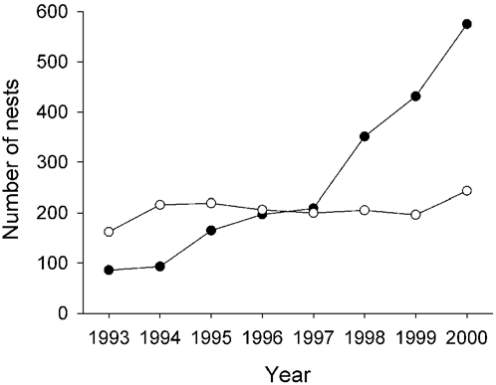
Growth of the Lesser kestrel study population from 1993 to 2000 in Sastago (the initial subpopulation, white dots; see Figure 1) and in the rest of the population (black dots).

The study population was founded in the 60s, benefiting from the very favorable habitat originated when the farmhouses where kestrels now breed were abandoned [21]. In 1993, when this study began, much of the population was still concentrated in a unique historical subpopulation named Sastago (Figure 1), which remained stable in size along the study period (Figure 2). However, from 1993 to 1997 the whole population experienced a steady population growth, with a much faster increase occurring from 1998 to 2000 (Figure 2). This resulted in a geographic expansion from the original Sastago subpopulation into new nearby areas (Figure 1). Here, we study the scaling properties of colony size variation through the years to show how these demographic and age-related behavioral characteristics occurring within the population relate with the dynamics of colony size frequency distributions.

## Results

**Figure 3:**
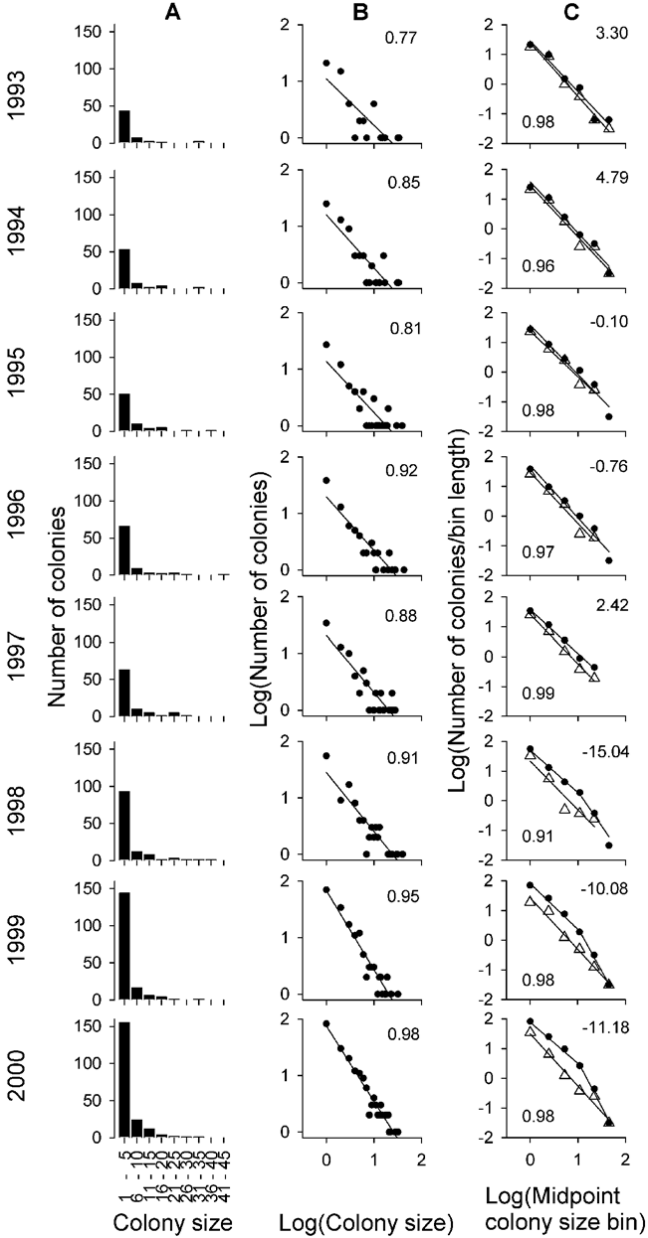
Frequency distribution of colony sizes across years in the Ebro Valley. A, Lineal histograms; x: colony size in lineal bins of five nests; y: frequency of colonies. B, Lineally binned log-log plots; x: no-binned (i.e. binned with bin length = 1) colony sizes, i.e. 1, 2, 3…; y: Log(frequency of colonies) i.e. 0 means 10^0^ = 1; 1 means 10^1^ = 10, etc. Inset numbers indicate the *R* ^2^ of the fit of each distribution to a power law. C, Multiplicative binned log-log plots for all the colonies studied (black dots), and only for the initial (Sastago, see Figure 1) subpopulation (white triangles); x: Log(midpoint of each bin). Because colonies are integers, the logarithmic midpoint was calculated as 10^(log(2n)+log(2n+1 − 1))/2^ where n is the number of the bin starting with 0, and the bins are in powers of two, i.e. 1–1, 2–3, 4–7, 8–15, 16–31 and 32–64 nests, so that the midpoint of the first three bins are 1, 2.449, 5.291; y: Log(mean number of colonies for each colony size within each bin), i.e. the number of colonies within a bin divided by the length of the bin calculated as 2^n^. Lower inset values indicate the *R* ^2^ of the fit of Sastago data to a power law. Best fits are also shown for the whole population; upper inset values indicate the difference in AIC between the power law and the truncated power law. Negative values denote a better fit of the truncated power law.

The frequency distribution of colony sizes during the study period was always highly right skewed (long-tailed) (Figure 3a). The use of lineally-binned log-log plots (i.e. bins = 1) suggested a power law distribution of colony sizes, particularly in the last study years, displaying a clear linear relationship between log(colony size) and their log(frequency) in the population (Figure 3b). Interestingly, however, this was only an artifact caused by non-binned plots (Figure 3b). Using appropriate multiplicatively-binned log-log plots [22], [23] we found that although between 1993 to 1997 colony sizes fitted well to a power law distribution (*R* ^2^ between 0.96 and 0.99), colony size variation in the last three years was better fitted to a truncated power law with a breakpoint at intermediate colony sizes (*ca*. ten pairs) (Figure 3c). This was supported by more than 10 points of AIC difference, so the addition of two parameters (the breakpoint and the slope of the second power law) was justified by the increase in fit (see methods).

On the basis of the previous information about the system [17]–[20], [24], [25] (see above), we hypothesized that this truncation could be the result of the population instability produced during population growth and expansion. Consequently, in the stabilized historical subpopulation (Sastago) (Figures 1, 2), this truncation should not occur. We thus analyzed separately the same dataset for Sastago colonies. As expected, we found a non-truncated power law during the eight study years in the Sastago subpopulation (Figure 3c). Thus, the truncation detected during the last three years of study was due to a disproportional larger accumulation of small colony sizes than of large ones in the new subpopulations (Figure 3).

## Discussion

Power laws are good descriptors of an array of object size frequency distributions in physical [26], human [27], [28], and biological systems [16], [29]–[31]. We have found here that power laws are appropriate tools to describe colony size variation. Interestingly, we have also found that dynamic deviations from this perfect scaling may help to identify underlying processes shaping colony size frequency distributions.

This is the first study to show the ontogeny of a truncation in the power law distribution of colony sizes throughout time in a wild population. This means that group size frequency distributions could be highly dynamic through years, not only in the characteristics of the breakpoint as previously reported [11], [13], but also changing from a power law to a truncated power law in a short time period. The appearance of this truncation at intermediate colony sizes, as a result of a largely unbalanced increase in the frequency of small colony sizes over large ones, suggested a link with the population instability observed during the eight-study period. The truncation becomes apparent from 1997 onwards (Figure 3), in parallel with the particularly rapid increase in population size during this period in the new subpopulations (Figure 2). The study population showed high levels of breeding success, but similar rates of adult survival compared to other populations of this species [21], [25], [32], indicating that the rapid population growth was mainly caused by an increase in the number of new recruits into the population (authors unpubl. data). Juvenile birds are known to possess poorer competitive skills when fighting to settle in medium-large colony sizes, thus becoming relegated to breed solitarily or in small colonies [18]. Moreover, they show the longest dispersal distances (from birthplace to first breeding colony) [19], [20]. Together, this suggests that in a situation of population growth and expansion, juveniles will play a major role on colony size dynamics. Thus, we predicted that the fast growing new subpopulations would be those producing the truncation by an unbalanced increase of small vs. large colony sizes. We successfully demonstrated this by showing that the historical subpopulation invariably exhibited a power law of colony sizes throughout the study period. Intriguing, the truncation of the power law occurred at those colony sizes (ca. ten pairs) at which the despotic behavior of adults starts playing a relevant repulsive force for new settlements [18], [24]. All large colonies were found to hold additional unused, but suitable nest sites [33], [34], ruling out the possibility that space constraints *per se* produced the truncation of the power law, and reinforcing the role of the despotic behavior in the dynamics of colony size variation in this population. Moreover, new small colonies, founded mainly by juvenile pairs, appear each year in the population, while large colonies reach a (dynamic) stable size [24].

**Figure 4:**
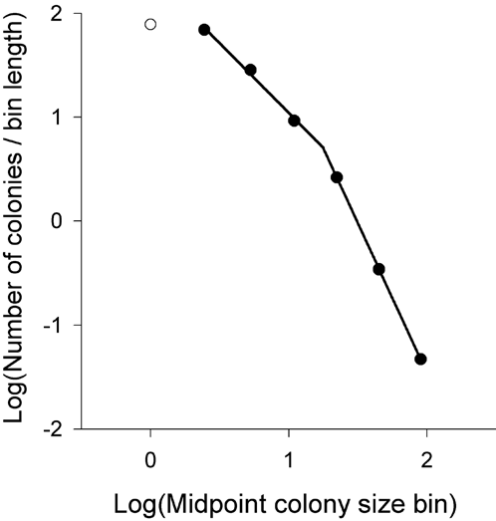
Frequency distribution of Lesser kestrel colony sizes in 1994 in the Guadalquivir Valley (SW Spain) (data from [33], [35]). Multiplicative bins in a log-log plot are used such as in Figure 3c. Colony sizes of one, that is, solitary nests, were not included in function fitting because we know that their frequency was underestimated. This is because of the low value of these solitary settlements for estimating population size (which was the aim of this survey) jointly with the high cost of searching for all of them because these are the most abundant and less conspicuous colony sizes. A truncated power law with a breakpoint at ca.18 pairs achieved a high fit to the data (*R* ^2^>0.9).

In a first attempt to explore the generality of our hypothesis in other systems, we examined snapshot data of colony size variation in another Lesser kestrel population from SW Spain. This was also an example of unstable population because it was undergoing a population decline at the time of the survey. In this population we also found a truncated power law distribution of colony sizes (Figure 4). The current literature suggests that large colonies are the first to suffer from population declines in this species [33], [36], because of density-dependent breeding performance during periods of low food supply [33]. This study area in SW Spain has experienced drastic agricultural intensification resulting in a deterioration of food resources for Lesser kestrels and a lowering of their breeding success [36]. Therefore, for these two separated populations, truncated power laws are associated with different types of population instability. However, further theoretical and empirical studies are needed to understand the mechanistic reasons by which power laws are dynamically truncated in an instability scenario, and to test the potential generality of this hypothesis in other animal populations.

This last study population also exemplifies the common problem which arises when interpreting truncated power laws from snapshot data, where it is difficult to ascertain whether small or large group sizes are the “missing” ones in the study system. In the Ebro Valley study population, thanks to a long-term monitoring program, we know that although the number of small colonies greatly increased across years, the number of large ones remained stable (Figure 3), creating a deficit of large colonies rather than of small ones. Thus, although snapshot data could be a first approach to identify underlying processes occurring in the system [11], we encourage long-term monitoring schemes in order to extract relevant information from them.

Our results shed light on a major methodological challenge in current bird coloniality research and suggest the need of improving the treatment of data in animal grouping research in general. Bird colony size frequency distributions, as in many other animal group sizes [37], are often reported to display long-tailed patterns when plotted in traditional histograms such in Figure 3a [38]. At present, this is most of the information we have about colony size variation in birds. However, these histograms are not very informative because most of the colonies gather together in the first bins (i.e. colony size intervals), and then, different long-tailed (right-skewed) distributions look similar in common histograms [22]. Differences between species, populations within species, or even temporal dynamics within populations, could be hardly captured with such an insensitive method. For instance, the ontogeny of a truncation shown in the multiplicatively binned log-log plots (Figure 3c) would have been impossible to detect from a direct inspection of the same data plotted in traditional lineal histograms (Figure 3a). It is true, however, that from Figure 3a it could be extracted that more small than large colonies were created, but it could not be appreciated the disproportionate increase leading to a truncation in the power law. Moreover, data from a single year plotted such as in Figure 3a could hardly suggest that something different is happening in small vs. large colony sizes, whereas it could be easily seen in the last plots of Figure 3c, or in the only plot available from the Lesser kestrel population in SW Spain (Figure 4).

An alternative approach is the use of lineally binned histograms in log-log plots (Figure 3b) (note that non-binned histograms are, in fact, linearly binned histograms with bins = 1). This plotting technique is being used in seminal papers about the scaling properties of animal group size frequency distributions [6], [11], [14]. However, we have found here contrasting results when exploring our data with this and multiplicative binned log-log plots (compare Figure 3b with c). Obviously, one approach must be wrong, and it has been demonstrated elsewhere that multiplicative binned log-log plots are much better for empirical long-tailed group size data [22], [23], [39; see Materials and Methods]. Thus, we encourage the use of multiplicative binned log-log plots for future works, while we suggest caution in the interpretation of these previous studies because erroneous results could be achieved [14], 23.

Patterns need to be *described* and *explained* in order to be *understood* [5]. We have found here that the study of the scaling properties of colony size frequency distributions allows an adequate formal *description* of colony sizes. For instance, we have found that in our case the distribution of colony sizes could be mathematically *described* using the few parameters of one or two power laws (i.e. *a* and *b* in *f(x) = ax^−b^*) (see below). This holds promise to also apply in other colonial bird species [15], thus allowing the statistical comparison of patterns that now could not be done because of the use of common histograms. Moreover, we have illustrated how we can use these patterns themselves as generators of hypotheses aimed to *explain* the mechanisms that are producing them. In our case, we have found that instability produced during population growth and expansion in a favorable habitat (population in NE Spain) or decline due to habitat degradation (population in SW Spain) can produce truncations on the scaling of colony size frequency distributions. This approach may prove useful for addressing important questions such as detecting population instabilities and forecasting their consequences in terms of grouping patterns. This is fundamental for management purposes of both pest and threatened species, so this approach also present practical applications that merit further study.

## Materials and Methods

### Nest distribution and population monitoring

When confronting the study of colonial bird populations, defining colony limits may be a non-trivial task because of the hierarchical spatial distribution of nests in nature [40]–[42]. In a previous study we found a fractal-like distribution of nests in White storks (*Ciconia ciconia*) [16], suggesting that the existence of colonies (as spatial units) should not be taken for granted. Consequently, our first task was to examine the scaling properties of nest spatial distribution in order to detect relevant scales at which breeding group size variation could be studied. To do this, we digitalized the location of all of the nests in the year when more nests were found (year 2000), and then performed a box-counting analysis [43]. This consisted on superimposing a series of grids, with increasing box side lengths, on the distribution map of nests in the population, and counting in each the number of boxes (squares) with at least one nest inside. Besides, we also calculated for each nest and for each occupied farmhouse the distance to the nearest nest or colony, respectively.

**Figure 5:**
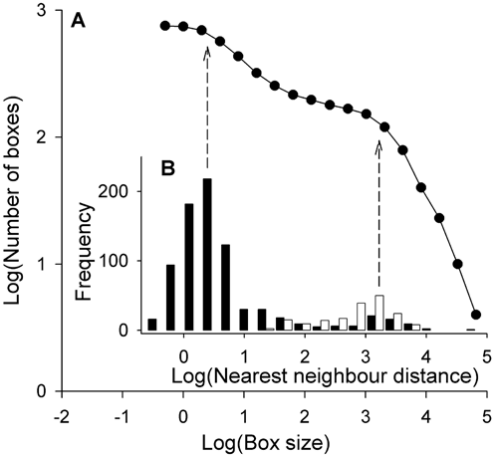
Scaling in the distribution of Lesser kestrel nests spatial distribution. A, Box-counting plot of the spatial distribution of nests in the most populated year in the Ebro Valley. B, Distribution of nearest neighbor distances between nests (black) and between colonies (white). All distances are in meters. Note the match between the mean distances and the inflection points (departing the pattern from a straight line, and thus from a fractal-like nest distribution pattern) in the box-counting plot. The small number of large between-nest distances corresponds to solitary nests for which the nearest nest is far away (in the closest colony).

The spatial distribution of nests clearly did not show a straight line (which would have indicated a fractal-like spatial distribution of nests), but had two clear inflection points, one at ca. 2 m and a larger one at ca. 2 km (Figure 5a), thus suggesting the existence of relevant scales in the distribution of nests. The smaller scale approximately corresponded to the average minimum distance among nearest nests (as indicated by arrows in Figure 5), whereas the larger one responded to the average distance between nearest farmhouses, thereby supporting the “colony” definition applied in early studies of this population [20]. Accordingly, we defined group size (colony size) as the final number of established pairs defending a nest-site in a single farmhouse in each breeding season. This parameter corresponds to the number of breeding pairs because of the predominant monogamous breeding system of this species.

In so doing, we applied a protocol accordant with the natural history of the species in the Ebro Valley. Lesser kestrels arrive to the study area from their sub-Saharan wintering (African) quarters from mid-February up to April. Thus, each year from 1993 to 2000, we conducted exhaustive surveys during these months in all farmhouses suitable for Lesser kestrels (i.e. with available cavities under the roofs) [34], we identified breeding birds by band lecture at distance, and we carried out behavioral observations from hides [18]. In addition from April to the end of July we monitored the breeding of all the breeding pairs through regular visits to the colonies, mapped the location of the nests, and banded some adults and almost all the chicks. Also during this period, we seized the opportunity to complete our survey by searching further for previously undetected new colonies created by late-breeders (mainly solitary first-breeders). Overall, we achieved a detailed and exhaustive survey of all the colonies which allowed us to obtain a precise knowledge of the total number of breeding pairs in each one.

### Plotting and functions fitting

A power law distribution could be expressed as *f*(*x*)* = ax^b^* where *f(x)* is the frequency of colonies with *x* nests (i.e. colony size), *a* is a constant indicating the intensity of the pattern, and *b* is the rate at which larger colony sizes become progressively less abundant (hence having a negative value). Power laws display a straight line when the log(*x*) is plotted against its log[*f(x)*], being the only functions that behave equally across scales (are scale-free or scale invariant). Note, that if we take logarithms at both sides of the equation, i.e. log[*f*(*x*)] = log(*a*)−*b* log(*x*), *f(x)* becomes a lineal function of *x* with a slope equal to *−b*.

We plotted colony size data in three different ways to achieve a complete picture of the pattern, and to compare our new approach to bird colony size variation against current practices in bird and animal grouping research. First, we used histograms as commonly done in the animal ecology literature [37], [44], [45]. Second, non-binned data was plotted in log-log plots, because this is a common technique used to plot cluster sizes [11], [14]. Finally, we represented the mean frequency of colony sizes for each bin in powers of two using log-log axis (see Figure 3 legend for details). The advantages of this kind of plots when detecting power laws are several and have been discussed elsewhere [22], [23], [39]. In brief, in these plots dots are homogenously distributed, placed on the correct geometric mean (given that we are plotting in a logarithmic axis), data is more homogeneously distributed within bins, and same weight is given along the range of *x*-axis values. Not doing so, often leads to report power laws (i.e. a linear relationship) where they are not. We fitted a power law in Figure 3b to exemplify this problem (e.g. compare black dots in Figure 3b *vs*. Figure 3c for year 1998–2000).

We fitted a power law function for each year in the appropriate Figure 3c. For the three last years with an apparent deviation from a power law (i.e from a straight line), we also fitted a truncated power law in two steps. First, we fitted a power law for the first three bins. Departing from the fitted value for the third bin, we fitted a second power law for larger bins. In this way, the truncated power law had two more parameters than the simple power law. We used AIC values to evaluate if the improvement in fit due to the flexibility allowed by the truncation justified the increase in two parameters. We accepted the truncated power law as a better model after imposing a restrictive cut-off of four AIC points.

## References

[1] Brown JH (1995). Macroecology..

[2] Sutherland WJ (1996). From Individual Behaviour to Population.

[3] Parrish JK, Hamner WM, Prewitt CT, Parrish JK, Hamner WM (1997). Introduction. From individuals to aggregations: unifying properties global framework and the grails of congregation. Animal Groups in Three Dimensions.

[4] May RM (1999). Unanswered questions in ecology. Philos Trans R Soc Lond B Biol Sci.

[5] Grimm V, Railsback SF (2005). Individual-Based Modeling and Ecology.

[6] Bonabeau E, Dagorn L, Fréon P (1999). Scaling in animal group-size distributions. Proc Natl Acad Sci U S A.

[7] Collett M, Despland E, Simpson SJ, Krakauer DC (1998). Spatial scales of desert locust gregarization. Proc Natl Acad Sci U S A.

[8] Theraulaz G, Bonabeau E, Nicolis S, Solé RV, Fourcassié V (2002). Spatial patterns in ant colonies. Proc Natl Acad Sci U S A.

[9] Amé J-M, Halloy J, Rivault C, Detrain C, Deneubeourg L (2006). Collegial decision making based on social amplification leads to optimal group formation. Proc Natl Acad Sci U S A.

[10] Buhl J, Sumpter DJT, Couzin ID, Hale JJ, Despland E (2006). From disorder to order in marching locusts. Science.

[11] Sjöberg M, Albrectsen B, Hjältén J (2000). Truncated power laws: a tool for understanding aggregation patterns in animals?. Ecol Lett.

[12] Solé RV, Goodwin B (2001). Signs of Life How Complexity Pervades Biology Basic Books New York.

[13] Lusseau D, Williams R, Wilson B, Grellier K, Barton TR (2004). Parallel influence of climate on the behaviour of Pacific killer whales and Atlantic bottlenose dolphins. Ecol Lett.

[14] Vandermeer J, Perfecto I (2006). A keystone mutualism drives pattern in a power function. Science.

[15] Schneider DC (2002). Scaling theory: application to marine ornithology. Ecosystems.

[16] Jovani R, Tella JL (2007). Fractal bird nest distribution produces scale-free colony sizes. Proc R Soc London B.

[17] Serrano D, Tella JL (2003). Dispersal within a spatially structured population of lesser kestrels: the role of spatial isolation and conspecific attraction. J Anim Ecol.

[18] Serrano D, Tella JL (2007). The role of despotism and heritability in determining settlement patterns in the colonial lesser kestrel. Am Nat.

[19] Serrano D, Tella JL, Forero MG, Donázar JA (2001). Factors affecting breeding dispersal in the facultatively colonial lesser kestrel: individual experience vs conspecific cues. J Anim Ecol.

[20] Serrano D, Tella JL, Donázar JA, Pomarol M (2003). Social and individual features affecting natal dispersal in the colonial lesser kestrel. Ecology.

[21] Tella JL, Forero MG, Hiraldo F, Donázar JA (1998). Conflicts between lesser kestrel conservation and European Agriculture Polices as identified by habitat use analysis. Cons Biol.

[22] Pueyo S (2006). Diversity: between neutrality and structure. Oikos.

[23] Pueyo S, Jovani R (2006). Comment on “A keystone mutualism drives pattern in a power function”. Science.

[24] Serrano D, Forero M, Donázar JA, Tella JL (2004). Dispersal and social attraction affect colony selection and dynamics of lesser kestrels. Ecology.

[25] Serrano D, Oro D, Ursúa E, Tella JL (2005). Colony size selection determines adult survival and dispersal preferences: Allee effects in a colonial bird. Am Nat.

[26] Turcotte DL, Rundle JB (2002). Self-organized complexity in the physical biological and social sciences. Proc Natl Acad Sci U S A.

[27] Batty M, Longley P (1994). Fractal Cities.

[28] Manrubia SC, Zanette DH, Solé RV (1999). Transient dynamics and scaling phenomena in urban growth. Fractals.

[29] Buldyrev SV, Dokholyan NV, Erramilli S, Hong M, Kim JY (2003). Hierarchy in social organization. Physica A.

[30] Brown JH, Grupta VK, Li B-L, Milne BT, Restrepo C (2002). The fractal nature of nature: power laws ecological complexity and biodiversity. Philos Trans R Soc Lond B Biol Sci.

[31] Pascual M, Roy M, Guichard F, Flierl G (2002). Cluster size distributions: signatures of self-organization in spatial ecologies. Phil Trans R Soc London B.

[32] Hiraldo F, Negro JJ, Donázar JA, Gaona P (1996). A demographic model for a population of the endangered lesser kestrel in southern Spain. J Appl Ecol.

[33] Tella JL (1996). Ecological Constraints Costs and Benefits of Coloniality in the Lesser Kestrel PhD Thesis.

[34] Forero MG, Tella JL, Donázar JA, Hiraldo F (1996). Can interespecific competition and nest site availability explain the decrease of lesser kestrels *Falco naumanni* populations?. Biol Cons.

[35] Hiraldo F, de la Riva M (1995). Colonias de Nidificación del Cernícalo Primilla (Falco naumanni) en Andalucía: Estado Actual de las Mismas Problemas de Conservación y Normas para su Manejo Convenio AMA-CSIC Sevilla Spain.

[36] Rodríguez C, Johst K, Bustamante J (2006). How do crop types influences breeding success in lesser kestrels through prey quality and availability? A modelling approach. J Appl Ecol.

[37] Krause J, Ruxton G (2002). Living in Groups Oxford Series in Ecology and Evolution Oxford.

[38] Brown CR, Stutchbury BJ, Walsh PD (1990). Choice of colony size in birds. Trends Ecol Evol.

[39] Pueyo S (2003). Irreversibility and Criticality in the Biosphere PhD Thesis.

[40] Berg Å, Lindberg T, Källebrink KG (1992). Hatching success of lapwings on farmland: differences between habitats and colonies of different size. J Anim Ecol.

[41] Arroyo B, Mougeot F, Bretagnolle V (2001). Colonial breeding and nest defence in Montagu's harrier (*Circus pygargus*). Beh Ecol Sociobiol.

[42] Ainley DG (2002). The Adélie Penguin..

[43] Halley JM, Hartley S, Kallimanis AS, Kunin WE, Lennon JJ (2004). Uses and abuses of fractal methodology in ecology. Ecol Lett.

[44] Brown CR, Brown MB (1996). Coloniality in the Cliff Swallow The effect of group size on social behaviour..

[45] Götmark F (1982). Coloniality in five Larus gulls: a comparative study. Ornis Scand.

