# Correction: Truncated Power Laws Reveal a Link between Low-Level Behavioral Processes and Grouping Patterns in a Colonial Bird

**DOI:** 10.1371/annotation/74fad966-c4c8-487c-a0fc-affb959ca508

**Published:** 2008-05-06

**Authors:** Roger Jovani, David Serrano, Esperanza Ursúa, José L. Tella

The Equation on the seventh line of the Figure 3 legend is incorrect. The legend with the corrected equation is available here:

**Figure 3:**
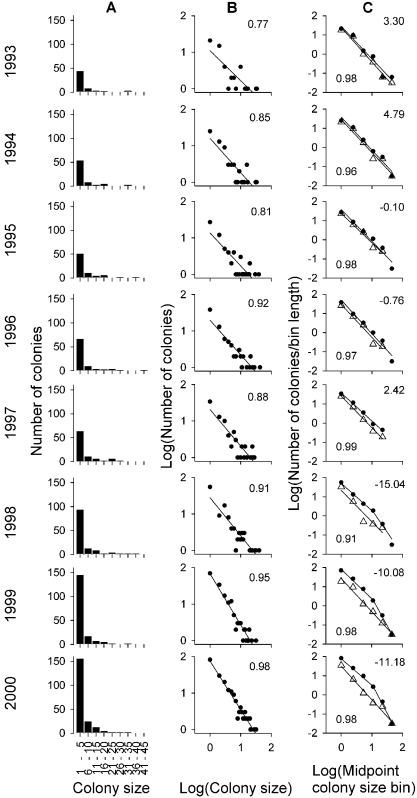
Frequency distribution of colony sizes across years in the Ebro Valley. A, Lineal histograms; x: colony size in lineal bins of five nests; y: frequency of colonies. B, Lineally binned log-log plots; x: no-binned (i.e. binned with bin length = 1) colony sizes, i.e. 1, 2, 3…; y: Log(frequency of colonies) i.e. 0 means 10^0^ = 1; 1 means 10^1^ = 10, etc. Inset numbers indicate the *R* ^2^ of the fit of each distribution to a power law. C, Multiplicative binned log-log plots for all the colonies studied (black dots), and only for the initial (Sastago, see Figure 1) subpopulation (white triangles); x: Log(midpoint of each bin). Because colonies are integers, the logarithmic midpoint was calculated as 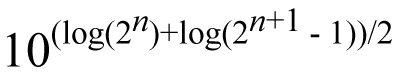 where *n* is the number of the bin starting with 0, and the bins are in powers of two, i.e. 1–1, 2–3, 4–7, 8–15, 16–31 and 32–64 nests, so that the midpoint of the first three bins are 1, 2.449, 5.291; y: Log(mean number of colonies for each colony size within each bin), i.e. the number of colonies within a bin divided by the length of the bin calculated as 2^*n*^. Lower inset values indicate the *R* ^2^ of the fit of Sastago data to a power law. Best fits are also shown for the whole population; upper inset values indicate the difference in AIC between the power law and the truncated power law. Negative values denote a better fit of the truncated power law.

