# Supplementary material for: Correction: Truncated Power Laws Reveal a Link between Low-Level Behavioral Processes and Grouping Patterns in a Colonial Bird
Source: PLoS One. 2008 May 6;3(5):10.1371/annotation/74fad966-c4c8-487c-a0fc-affb959ca508. doi: 10.1371/annotation/74fad966-c4c8-487c-a0fc-affb959ca508 (PMC2556497; doi:10.1371/annotation/74fad966-c4c8-487c-a0fc-affb959ca508)
Supplement: Supplementary file 1 [file pone.74fad966-c4c8-487c-a0fc-affb959ca508.s001.pdf]

PLoS PUBLIC LIBRARY of SCIENCE

Figure 3. Frequency distribution of colony sizes across years in the Ebro Valley.

A, Lineal histograms; x: colony size in lineal bins of five nests; y: frequency of colonies. B, Lineally binned log-log plots; x: no-binned (i.e. binned with bin length=1) colony sizes, i.e. 1, 2, 3…; y: Log(frequency of colonies) i.e. 0 means $10^0 = 1$; 1 means $10^1 = 10$, etc. Inset numbers indicate the R^2 of the fit of each distribution to a power law. C, Multiplicative binned log-log plots for all the colonies studied (black dots), and only for the initial (Sastago, see Figure 1) subpopulation (white triangles); x: Log(midpoint of each bin). Because colonies are integers, the logarithmic midpoint was calculated as $10^{(\log(2^n)+\log(2^{n+1} - 1))/2}$ where n is the number of the bin starting with 0, and the bins are in powers of two, i.e. 1-1, 2-3, 4-7, 8-15, 16-31 and 32-64 nests, so that the midpoint of the first three bins are 1, 2.449, 5.291; y: Log(mean number of colonies for each colony size within each bin), i.e. the number of colonies within a bin divided by the length of the bin calculated as 2^n. Lower inset values indicate the R^2 of the fit of Sastago data to a power law. Best fits are also shown for the whole population; upper inset values indicate the difference in AIC between the power law and the truncated power law. Negative values denote a better fit of the truncated power law.
